# Fight the Fire: Association of Cytokine Genomic Markers and Suicidal Behavior May Pave the Way for Future Therapies

**DOI:** 10.3390/jpm13071078

**Published:** 2023-06-29

**Authors:** Xenia Gonda, Gianluca Serafini, Peter Dome

**Affiliations:** 1 Department of Psychiatry and Psychotherapy, Semmelweis University, 1085 Budapest, Hungary; dome_peter@yahoo.co.uk; 2 NAP3.0-SE Neuropsychopharmacology Research Group, Hungarian Brain Research Program, Semmelweis University, 1085 Budapest, Hungary; 3 International Centre for Education and Research in Neuropsychiatry (ICERN), Samara State Medical University, 443079 Samara, Russia; 4 Department of Neuroscience, Rehabilitation, Ophthalmology, Genetics, Maternal and Child Health (DINOGMI), Section of Psychiatry, University of Genoa, 16126 Genoa, Italy; gianluca.serafini@unige.it; 5 IRCCS Ospedale Policlinico San Martino, 16132 Genoa, Italy; 6 National Institute of Mental Health, Neurology and Neurosurgery, 1135 Budapest, Hungary

**Keywords:** suicide, neuroinflammation, cytokine, biomarker, genomic marker, precision psychiatry

## Abstract

The fight against suicide is highly challenging as it may be one of the most complex and, at the same time, most threatening among all psychiatric phenomena. In spite of its huge impact, and despite advances in neurobiology research, understanding and predicting suicide remains a major challenge for both researchers and clinicians. To be able to identify those patients who are likely to engage in suicidal behaviors and identify suicide risk in a reliable and timely manner, we need more specific, novel biological and genetic markers/indicators to develop better screening and diagnostic methods, and in the next step to utilize these molecules as intervention targets. One such potential novel approach is offered by our increasing understanding of the involvement of neuroinflammation based on multiple observations of increased proinflammatory states underlying various psychiatric disorders, including suicidal behavior. The present paper overviews our existing understanding of the association between suicide and inflammation, including peripheral and central biomarkers, genetic and genomic markers, and our current knowledge of intervention in suicide risk using treatments influencing inflammation; also overviewing the next steps to be taken and obstacles to be overcome before we can utilize cytokines in the treatment of suicidal behavior.

## 1. The Impact of Suicide

The fight against suicide is highly challenging as it may be one of the most complex and, at the same time, most threatening among all psychiatric phenomena [1]. Furthermore, while psychiatry, in general, for several reasons, significantly lags behind other medical specialties when it comes to precision medicine [2], a precision approach for prediction, prevention, and intervention in suicidal behavior is, although necessary, an even bigger challenge [3]. Suicide may be considered a phenomenon that needs to be predicted in the near or not-so-near future rather than at present. However, it is highly heterogeneous, and rather than being a distinct category, it is a phenomenon arising as part of various neuropsychiatric disorders and with multiple neurobiological pathways and mechanisms underlying its etiopathology [1].

Suicidal behavior is still ranked among the first ten preventable causes of death worldwide [4], with stable or even increasing rates in several countries during the past 50 years, although extensive scientific and clinical efforts [5] have been carried out to understand its emergence and attenuate its risk. Every year at least 700,000 lives are lost due to suicide [6], meaning one suicidal death every 40 s; thus, there are more people dying from suicide than as a consequence of homicide and war [7]. Even more alarmingly, suicide in adolescents and young adults is the fourth leading cause of mortality in subjects aged 14–29 worldwide [6], and it is the second leading cause in this age group in developed countries (https://www.nimh.nih.gov/health/statistics/suicide) [8]. Despite these shockingly high numbers, these figures likely underestimate the real prevalence, as suicides are underreported due to societal taboos and criminalization of suicidal acts in several cultures and societies. Furthermore, the burden and impact of suicide are not constrained to those dying from it, as for every suicidal death, there are at least 10–40 suicide attempts, and an even higher number of patients experiencing suicidal ideation [4,9]; suicidal behavior of these subjects is associated with significant distress and suffering. In addition, all forms of suicidal behavior exert adverse impacts on family and friends, which makes suicide a major contributor to the profound impact on mental health and is generally a critical issue in mental healthcare. Finally, globally self-harm is responsible for 1.26% of disability-adjusted life years (DALY) [10].

## 2. Unmet Needs in Our Current Approach to Predict Suicidal Behavior

In spite of its huge burden and advances in neurobiology research, understanding and predicting suicide remains a major challenge for both researchers and clinicians. While a number of clinical, psychosocial, and sociodemographic risk factors and prognostic indicators for suicidal behavior [11] have been identified in the past decades, they are of limited clinical relevance due to their low predictive value [12] and their generally non-modifiable nature. To understand why it is difficult to predict suicidal behavior accurately utilizing risk assessment tools, we need to keep some reasons in mind. For instance, due to the relative rarity of suicide, some of these reasons are of a statistical nature. In addition, suicidal subjects often do not reveal their suicide ideations/intentions during a clinical assessment [1].

Thus, to actually be able to identify those patients who are likely to engage in suicidal behaviors and identify suicide risk in a reliable and timely manner, we really need more specific, preferably behavioral and biological markers/indicators to develop better screening and diagnostic methods. To achieve this goal, more objective markers, especially biomarkers, potentially reflecting modifiable processes, are imminently required to improve prediction, risk screening and assessment, as well as develop efficient methods for prevention and intervention targeting processes represented by these biomarkers [13].

In order to achieve these goals, first and foremost, we need to gain a better insight into the neurobiological processes underlying suicidal behavior to offer a novel understanding of the pathophysiology of suicide and identify new treatment targets and approaches [14]. The necessity of this task, as well as our currently shifting approach to suicide, is also reflected by the fact that in the DSM-5, suicide is increasingly considered an independent phenomenon with a specific biological vulnerability and not just an outcome of various psychiatric disorders. In fact, suicide behavior disorder (SBD) was introduced in DSM-5 as a disorder for further consideration and potential acceptance into the diagnostic system [15].

## 3. The Complex, Multicausal, and Heterogeneous Nature of the Suicide Phenomenon

Understanding the neurobiological background of suicide is still challenging for several reasons. One of the reasons is that suicide is a very heterogeneous phenomenon, both concerning its manifestation and its neurobiological background [16,17]. Suicidal behavior is highly complex and multicausal and cannot be associated with either a single gene, biological dysfunction, specific cause, or a single type of stressor; rather, there is a large number of distal and proximal factors contributing to its emergence. Distal factors usually include genetics, personality traits, such as impulsivity or aggression, early traumatic events, and neurobiological dysfunctions, while proximal risk factors may include somatic or mental disorders as well as acute or current stressors and psychosocial crises [1,18]. These two major groups of contributors form a complex interaction, as described by the diathesis–stress model of suicidal behavior, explaining the interaction between trait-like biological and personality-based susceptibility and external or internal stressors acting as triggers [18,19]. In order to understand and predict who is at increased risk of suicide, we need to identify biomarkers associated with the above contributors or risk factors of suicidal behavior [13].

As the risk factors identified up to date have not been proven to be useful in predicting suicide in everyday clinical settings, and we have not noticed much improvement in this specific regard in the past few decades [5,20], a profound conceptual change is warranted. Such a change should embrace novel approaches, such as machine learning algorithms and new technologies, including novel biomarkers and other objective indicators. This conceptual change needs to focus on individual patients instead of predicting suicide risk based on demographic factors and stratification.

A recent review of the most important predictors of suicidal behaviors pointed to two categories, one including imaging findings and the other biochemical and genomic findings [18] and concluded that biomarkers with the best clinical potential for prediction of different types of suicide risk include indices related to decreased serotonin function, neuroplasticity, lipids, and finally inflammation, the topic of the current paper [18,21].

## 4. Conceptualization of Suicide: The Role of Internal and External Contributors

Traditionally, different manifestations of suicidal behavior may be conceptualized along a continuum or spectrum spanning from suicidal ideation, plans, suicide gestures, attempts, and aborted suicide to completed suicide. This continuum approach towards the distinction between types of suicidal behavior is based solely on the severity of intent to die or lethality and violence of the method involved in the suicidal behavior. However, as mentioned before, considering that suicide is a highly heterogeneous and complex phenomenon in its main manifestation, different types of suicidal behavior may not be treated as a continuum, and there may be different etiological processes involved at the neurobiological level [22].

Different types of suicidal behavior may be differentiated by aspects other than lethality. For instance, the involvement of internal/endogenous/genetic/neurobiological versus the involvement of external/environmental factors may be another differentiating aspect, which may help us to understand not only the phenomenology but even the neurobiology of suicide. Of course, both neurobiological and environmental contributors are present in the majority of suicidal behaviors; however, their relative weight may show marked differences [1]. Furthermore, reactivity to environmental events and triggers resulting in suicide are also influenced by genetic and neurobiological factors; however, these events are likely to be greatly different from those genetic and neurobiological pathways, which directly underlie suicidal behavior.

Several forms of suicidal behavior may be associated with a stronger contribution of genetic and neurobiological determinants and with a relatively lesser effect of actual life events or stress-related triggers [1]. For instance, it appears that suicidal behavior associated with major mental disorders such as bipolar disorder, major depressive disorder or schizophrenia, as well as severe somatic illnesses, especially where activation of the immune system is involved, or somatic disorders treated with certain medications, such as immune modulators, may have a predominance of neurobiological determinants and may have a higher risk of occurring in the absence of adverse environmental events. In other forms of suicidal behavior, environmental triggers will carry a bigger weight, such as suicidal behavior associated with narcissistic injuries, severe interpersonal and other difficulties in personality disorders or suicide following major acute or recent stresses or crises. The key aspect underlying all the different forms of suicidal behaviors seems to be the divergent etiological processes underlying these different manifestations of suicidal behavior since screening, prevention and treatment will mainly focus on these pathways. In the case of more exogenously induced suicidal behaviors, risk and prediction biomarkers may be more related to behavioral, cognitive, emotional, and personality variables, and treatment may need to focus more on reactivity towards the environment and life events, possibly with psychotherapy playing a bigger role. Conversely, in the case of more endogenously determined suicidal behaviors, we should identify risk and predictive biomarkers and treatment targets related to the possible multifaceted underlying biological processes and therefore rely more on psychoactive medications for treatment.

However, at this point, we do not have available specific medications to control suicidal behaviors in clinical practice. Drugs currently used to decrease suicide risk and prevent attempts and completed suicide almost exclusively target the psychiatric disorders associated with increased suicide risk, especially mood disorders and associated symptoms [23]. These drugs are not specific for suicidal behavior, and in many cases, their effectiveness is also questionable [1]. Therefore, we need to better understand the divergent neurobiology of suicide to find adequate biomarkers for screening purposes and identify neurobiological processes as more specific targets for more effective interventions; the latter is especially needed for those cases which are less accessible for psychotherapy for prevention and intervention.

## 5. A Novel Approach to Understanding Suicidal Behavior: The Role of Neuroinflammation

One such potential novel approach is offered by our increasing understanding of the involvement of neuroinflammation based on multiple observations of increased proinflammatory states underlying various psychiatric disorders, including suicidal behavior [24].

We have long understood that neuroinflammation plays a primary pathophysiological role in various chronic somatic illnesses, which had not been hypothesized to be predominantly related to immune activation or immunological disorders, such as cardiovascular illnesses or oncological disorders. In spite of a longstanding view that the central nervous system is isolated by the blood–brain barrier (BBB) from the peripheral immune system, a recent paradigm shift paved the way towards the recognition of multiple layers of immune surveillance, even in the brain [25]. Now it is accepted that cytokines produced in the periphery may permeate the BBB and thus convey signals to the central nervous system. It is also now well-known that cytokines are secreted locally in the brain by activated microglia, astrocytes as well as endothelial cells. Furthermore, we are also aware of the key role of cytokines in contributing to the development as well as maintenance of central nervous system functions [26,27].

Similarly, we increasingly recognize the presence of inflammatory alterations in multiple psychiatric disorders and the potential participation of the immune system in at least subgroups of psychiatric disorders and symptoms across diagnoses among selected psychiatric patients [28].

There is also an increasing number of studies supporting the role of the immune system and neuroinflammation in different psychopathological manifestations and even specifically in suicidal behavior. The initial observations sparking interest described a markedly elevated risk of depression and self-violence in toxoplasma infections [29,30], as well as increased suicidal ideation in patients treated for various conditions such as hepatitis B, hepatitis C, HIV, melanoma, or multiple sclerosis with biological therapies involving interferon-α (IFN-α) and interferon-β (IFN-β) [31,32,33,34]. Other lines of evidence are related to various somatic illnesses underscored by extended immune system activation, such as autoimmune conditions or allergies, and including multiple sclerosis, lupus or asthma [35,36,37,38,39,40,41], where the increased risk of mood disorders and suicidal behavior were not associated with the burden and distress but were simply linked to having a chronic and debilitating somatic condition. Finally, the relatively recently discovered link between gut microbiota and depression is also probably mediated by inflammation in addition to several other mechanisms. Recently, an enterotype (a gut microbiota biomarker based on clusters of bacterial communities living in the gut) was found to be associated with suicidal behavior [42]. These initial findings were followed up by various clinical studies specifically focusing on the association between different aspects of suicidal behaviors and different inflammatory parameters. For instance, a seminal study reported the existence of an association between increased cerebrospinal fluid IL-6 levels in recent suicide attempters, which also correlated with the severity of depression [43], and another study found decreased levels of anti-inflammatory and neuroprotective IL-8 in suicide attempters when compared to healthy controls, pointing to the role of immune dysregulation in suicidal patients [44]. A comprehensive meta-analysis also showed that IL-6 and IL-1β plasma and CSF levels are able to robustly distinguish suicidal victims from nonsuicidal patients and healthy controls [45]. Other studies reported a positive association between neuroinflammation and suicidal ideation intensity among psychiatric patients [35], and a correlation between suicidal ideation in patients with major depressive disorder even after controlling for the severity of depression and active attempts [46]. Post-mortem studies found increased IL-4 and IL-13 mRNA levels in the orbitofrontal context of suicidal victims [47], increased Il-1β, IL-6, TNF-α mRNA and protein levels in teenage victims’ cortices [48], as well as a higher proportion of activated microglia in the anterior cingulated cortex (ACC) of depressed suicide victims when compared to subjects without psychiatric disorders dying from other causes [49]. A specific microgliosis reflecting increased inflammation in depressed and schizophrenic patients committing suicide has also been observed [50]. CRP levels have also been frequently implicated in association with suicidal ideation and behaviors [51,52,53], and a recent meta-analysis confirmed a significant association between increased CRP and any type of suicidal behavior when compared to both nonsuicidal depressed patients and healthy controls [54].

These studies point to the direction of an association between neuroinflammation and suicidal behavior, which specifically characterizes suicidal behavior rather than being related to the underlying psychiatric illness, such as major depression. A clinically more useful attempt is the identification of a panel of variants, rather than single ones, to predict suicide risk in a more comprehensive approach. For instance, parallel alteration of peripheral levels of cytokines, such as IL-6 and IL-8, WBC count, and polymorphonuclear leukocyte count were associated with the risk of suicidal ideations and attempts in females compared to depressives with no ideation have been reported [55]. A panel of 23 inflammatory markers was also able to identify a significant association between increased neuroinflammation and suicide risk [56].

Prospective studies also support this association showing a four-times higher risk of completed suicide in those with CRP levels higher than 3 mg/L indicative of inflammation compared to those with CRP levels below 1 mg/L [57]. Higher baseline TNF-α levels were reported to predict worsening suicidal ideation after 12 weeks of follow-up [58]. Meta-analyses, for example by reporting a robust association between IL-6 and IL-18 levels and suicidality, also conclude that there may be an inflammatory signature in suicidal behavior which is likely independent of the primary diagnoses and underlying conditions and is really characteristic of suicidal behavior and risk [45].

## 6. An Evolutionary Perspective on The Role of Neuroinflammation in Psychiatric Disorders and Suicide

In order to understand the role of neuroinflammation in the background of suicidal behavior, besides the association between inflammatory mediators and suicidal behavior, it is also important to understand what the exact link between immune activation and suicide may be. Indeed, the association between immunological activation and different central nervous system conditions, such as depression or self-destructive behavior, may have been advantageous from an evolutionary perspective [25]. Throughout human evolution, and in fact, up to very recent periods, the major cause of death was injuries and related infections, meaning that from an evolutionary point of view, there has been strong selective pressure for genetic variants enhancing immune functions and mechanisms related to host defense. As a result of this, microbial infections have been conceptualized as being among the primary drivers of human evolution [59]. As cytokines and immunological mediators were able to enter the brain, they also impacted the central nervous system, triggering what we call sickness behavior [60], which is an adaptive response resembling depression, characterized by anhedonia, behavioral inhibition, anxiety, loss of appetite and weight, and somatic symptoms such as fatigue, malaise and hyperalgesia [60,61]. Similar to immune activation in the periphery, brain sickness behavior served the purpose of triggering subjective states, emotions, cognitions and behavior, which maximized the risk of survival and minimized the chance of actions that would have increased the risk of death, thus decreasing motivation, exploration, interest in the environment or social encounters [59,62]. Taken together, abnormal inflammatory activation and its impact on the brain promoted the odds of survival in highly pathogenic environments, where stress meant physical stressors leading to physical injury. Therefore, stress-relevant neurocircuits and the immune system formed an integrated system working in parallel in order to protect the organism from physical threats and pathogens [25]. Currently, however, the modern world is characterized by both a smaller risk of infections due to more sanitized environments and a smaller risk of physical injuries. Modern world stressors, rather than the risk of physical injuries, are mainly related to psychosocial events, which are perceived as being just as threatening. This way, thanks to the integrated system, this usually prepares us for the expectation of injuries, wounds, and infection, activating immunological and inflammatory pathways and leading to an elevated level of circulating proinflammatory cytokines, which, even in the absence of actual injury or infection, still fine-tune the central nervous system triggering behaviors related to sickness behavior and resembling depression, decreasing mental well-being [25]. Considering that we are facing chronic psychosocial stress, it may be paralleled by a chronic, although mild, elevation of circulating proinflammatory cytokines. In addition, several other environmental risk factors associated with mental health conditions, including depression and suicidal behavior, early adversities, obesity, and a diet high in processed foods, are also proinflammatory in their main nature. This association between stress-associated psychiatric symptoms, specific disorders and neuroinflammation is also supported by the fact that in most recent genome-wide association studies (GWAS), as well as other genetic studies regarding depression, the best-replicated alleles are those associated with proinflammatory processes or behaviors that reduce pathogen exposure [63,64,65].

## 7. The Grey Eminences of Neuroinflammation: The Role of Cytokines in Psychiatric Conditions and Suicide

Understanding different neurobiological mechanisms underlying the increased risk of suicide is an important step to developing better, more accurate, and effective ways for predicting, screening, and managing suicide risk. Importantly, risk factors of suicide include familial, developmental, environmental, social, psychological, somatic, and medical contributors, as well as genetic variants and epigenetic factors. While these contributors, in most cases, generally characterize patients with psychological disorders, especially depression, and lack the necessary specificity for suicidal behavior, it appears that many of these clinical conditions may exert their effect by enhancing proinflammatory cytokine production and signaling. Thus, rather than abandoning the traditional risk factors in state-of-the art models of suicide and its management, the next step warrants a better understanding of how these phenomena are associated with or mediated by alterations in the inflammatory processes involved in predisposing to suicide or precipitating suicide risk. In other words, the next step would be to identify reliable biomarkers for suicidal risk, which could be used to detect approaching suicidal behavior in those who are faced with or present with traditional risk factors.

The most relevant evidence on the immunological association of suicidal behavior focuses on cytokines, a heterogeneous group of regulatory polypeptides released by immunocompetent cells, including macrophages and lymphocytes. Cytokines play an important role in host defense against pathogens and in repairing tissues after injuries [66]. As mentioned before, cytokines produced in the periphery are able to cross into the brain by active transport via the BBB; furthermore, they are also produced in the central nervous system by neurons, astrocytes, and activated microglia [67]. In addition to defending the organism against pathogens, they are increasingly recognized as messenger molecules in the central nervous system, and, beyond participating in neuroinflammatory processes, they may possess neurotransmitter-like properties. Cytokines are also involved in the modulation of neuroendocrine function, play a role in neurodegenerative processes, and have been implicated in neurodevelopmental disorders as well as in the neurobiology of stress-related conditions, such as major depression and suicide [26,27,68,69]. Proinflammatory cytokines, such as IL-6, IL-1, and TNF-α, appear to be the most relevant in this sense, considering their actions in the brain as one of their most remarkable and relevant characteristics is their pleiotropy to bind to different cell target types [25,67,70].

Cytokines may be involved in impacting central nervous processes, including emotions, cognitions and behaviors, in multiple ways and via various mechanisms, both directly and over a prolonged period. Cytokine receptors have been found in several brain regions involved in major depression and suicidal behavior; thus, cytokines are able to directly influence neuronal functions in these areas inducing specific effects on behavior and emotions [28,66,71]. Cytokines may also modulate the concentration, function and signal transduction of monoamine neurotransmitters, especially serotonin and its metabolites, in various brain regions implicated in the pathophysiology of suicidal behavior, including the prefrontal cortex, hippocampus and amygdala [72,73,74,75,76]. Furthermore, cytokines may also induce changes in the function of the HPA-axis, the other major system implicated in suicidal behavior [74,77]. Neuroinflammation and increased cytokine levels are also associated with the activation of the kynurenine pathway, inducing the downstream production of metabolites with effects on glutamate neurotransmission [78,79,80,81,82]. Via the above-mentioned mechanisms, cytokines are more likely to exert an influence even on fetal and later neurodevelopment. Immune mediators and proinflammatory cytokines are associated with suicide-related phenotypes on both state and trait levels.

As mentioned before, proinflammatory cytokines acutely induce sickness behavior [83] in order to alter emotions, cognitions, and behavior in the direction that could best help cope with neuroinflammation, injury or infections, increasing the chance of survival and decreasing further exposure to risk. This includes lethargy, major depression, cognitive changes, such as failure to concentrate, anorexia, sleep disturbances, decreased personal hygiene, and social withdrawal. This is also reflected in experimental contexts in which, for example, cognitive changes related to suicidal behavior, such as changes in verbal memory, cognitive speed, or executive functions, occurred together with altered cytokine levels [83]. In addition to their acute effect, even on the level of personality traits and temperaments, there are several manifestations that are related to an increased risk of suicidality and are also associated with increased levels of proinflammatory states and cytokines, including IL-6 and CRP, such as neuroticism and harm avoidance [84,85,86], increased hostile, angry or aggressive traits [87,88,89], hopelessness and pessimism [90,91,92,93,94]. Evidence suggests that circulating proinflammatory cytokines may significantly affect mood states in both the short- and long-term periods via functional alterations of limbic circuits. Ultimately, cytokines cause the disruption of regulatory cortico-striatal circuits triggering abnormalities of executive function, emotional regulation, hopelessness, and impulsivity, contributing to suicide vulnerability and at-risk conditions. Patients with suicidal vulnerability might thus respond to social rejection or other psychosocial stressors with abnormal inflammatory activity and psychological pain, a process triggered by adverse social experiences and having activated sensitized pathways [23,35,68].

## 8. From Understanding of Ancestral Mechanisms to Modern Clinical Practice: How Can Neuroinflammation Be Exploited for Better Management of Suicide Risk?

While we need deeper knowledge to exactly understand the inflammatory underpinnings of suicidal behavior, we are still left with the question of how we can put this accumulating insight and knowledge toward advancing the handling of suicidal behavior in clinical practice. When we discuss the correct management of suicide and suicide risk, there are two major things we strive for: the first one is prediction and screening; the other is identifying novel targets and more efficient methods for intervention and prevention.

Our current strategy of predicting suicide risk is based on obtaining clinical and sociodemographic data via medical records; identifying major life events as reported by the patients or collaterals; using different self-reported instruments to identify suicidal thoughts, state-related factors such as depression or anxiety, or temperamental or personality traits related to suicide risk; or conducting thorough and lengthy clinical interviews. This approach has several drawbacks because it collects subjectively recalled and subjectively evaluated data, and it is a very lengthy and laborious process, and often of low specificity and a low predictive value. Therefore, in order to improve the accuracy of screening and prediction, we need objective biomarkers and indicators, such as those related to neuroinflammation or other neurobiological markers. This, especially considering that suicide develops as a result of a multicausal process where the dysregulation of the immune system is only one of the multiple causal factors, would not necessarily mean abandoning our current knowledge on suicide, but it stresses the need to incorporate novel biomarkers into a multistage system for prediction, prevention, and treatment of suicide [67].

## 9. Cytokine and Other Immune-Related Markers of Suicidal Behavior

While alterations in cytokine levels or cytokine gene expression have been reported in the brain of suicidal victims, such samples are accessible only post-mortem; thus, for purposes of screening, easily accessible and readily measurable peripheral cytokines would be absolutely necessary.

Cytokines were, in fact, proposed as blood biomarkers associated with suicide risk several years ago [95]. Furthermore, not only singular cytokines but multidimensional inflammatory markers [68] or an inflammatory index comprising the most relevant peripheral proinflammatory cytokines, including CRP, IL-6, IL-10 and TNF-α, have been found to predict those depressed patients who also manifest suicidal ideation [46]. A meta-analysis reported significant elevation of IL1-β and IL-6 as well as lower IL-2 levels among suicidal patients compared to non-suicidal depressed patients as well as healthy control subjects [45], and a retrospective cohort study also reported that decreased IL-2 levels may predict completed suicides in previous attempters [44]. CRP levels were associated with a history of suicide attempts but not with current ideation, suggesting its potential role as a trait marker for suicidal behavior, with a marked risk of suicide in those with a CRP level higher than 3 mg/L [96]. VEGF blood levels were also shown to be associated with suicidal ideation and intent [97].

Although more invasive and more difficult to access and assess than plasma levels, CSF cytokine levels have also been associated with different types and parameters of suicidal behavior, for instance, in a study evaluating levels of 20 different potential mediators in suicide attempters, three factors differentiating patients with respect to risk of suicide were identified. Factor 1, including eotaxin, eotaxin-3, MCP-4 (human monocyte chemoattractant protein-4), MDC (macrophage-derived chemokine) and TARC (thymus and activation-regulated chemokine) showed a significant association with severe depression without suicidal behavior; factor 2, including proinflammatory cytokines IL-6, IL-8 and TNF-α, was associated with severe depression coupled with suicidal ideation and intent; whereas factor 3, including IL-6, orexin as well as the neurotransmitter metabolites 5-HIAA (5-hydroxy-indoleacetic acid) and HVA (homovanillic acid), showed an association with a violent suicide attempt and completed suicide [98].

As for brain markers associated with completed suicide, research suggests that both brain-originating cytokines leaking to the periphery and cytokines of peripheral origins crossing into the brain via the BBB may both be involved in suicidal behavior [68]. The central nervous system of psychiatric and suicidal patients may be more exposed to peripherally produced cytokines due to the potentially compromised function of the BBB. Disrupted BBB function may be, in fact, a consequence of ongoing immune activation of either a peripheral or central origin, which, by allowing an even larger influx of cytokines into the brain, may lead to a positive feedback or feed-forward-like inflammatory mechanism in suicidal behavior [68]. There are several studies describing microglial activation in suicide victims’ brain tissues, reflecting chronic low-grade cerebral neuroinflammation across different diagnoses, which, via microglial activations, may have a long-term impact on neuronal development as well as function via increasing cytokine production in the brain, which in turn impacts serotonin and noradrenaline neurotransmission known to be involved in suicidal behavior [50,68]. Several studies also reported increased expression of cytokines, including IL-4, IL-13, IL-6, IL1-β, TNF-α, and IL-10, in specific brain regions of suicidal victims known to be associated with different suicidal behaviors, such as the orbitofrontal cortex, parietofrontal cortex, etc. [47,48,49].

## 10. The Role of Cytokines and Related Biomarkers in Predicting Suicide Risk following Proximal and Distal Stressors

As their production is at least partially related to the perception of stress, proinflammatory cytokine levels may actually mediate the effect of different types of environmental stressors in suicide risk in line with the prevailing diathesis–stress model of suicidal behavior. This model postulates that there is a specific vulnerability developing based on genetic, neurobiological, and early environmental factors, which are triggered by actual stressors later on [66]. Variation in cytokine genes or increased vulnerability towards chronic low-grade inflammation and elevated proinflammatory cytokine levels, especially in response to chronic psychosocial stress, may be an important contributor toward suicide risk. Stress is known to lead to divergent consequences via disturbing HPA-axis function, a dysregulation also implicated in suicidal behavior [99]. HPA-axis disruption is also associated with altered cytokine levels [100], besides other relevant changes. Considering that the majority of suicide victims experience exposure to stressful life events in the year preceding their suicidal act [101] and the association between stress, neuroinflammation and suicide, it would be crucial to find out if there are genetic variants predisposing to a higher risk of a neuroinflammatory response in response to stress, also in parallel to psychological factors increasing stress reactivity possibly related to suicide. For instance, negative effects triggered by social rejection show an association between increased inflammatory response [102], and several studies have also demonstrated a link between social isolation and the activation of inflammatory processes [103,104,105], paralleled by an increase in the expression of genes encoding proinflammatory immune responses in lonely people exposed to negative social experiences [68,105,106].

Importantly, not only acute or current psychosocial stress may lead to HPA dysfunction, but early childhood traumatic events may even cause long-term dysregulation and chronic proinflammatory states [107], including altered proinflammatory cytokine and CRP levels [108], and immune dysregulation has been shown to be associated with suicide attempts and deaths in adulthood [109]. It would also be crucial to understand both long-term neuroinflammatory predictors of suicide risk and what genetic variants increase the risk of long-term neuroinflammation and, consequently, later suicidal behavior in case of exposure to early traumas and stressors [110].

## 11. Inflammatory and Cytokine Genetic Markers of Suicidal Behavior

Family studies estimate the heritability of suicidal behavior to be between 17 and 55% [111], while SNP-based heritability of suicidal behavior is about 7.6% for suicidal thoughts and behaviors [112]. Until recently, few studies specifically investigated the association between variation in inflammation-associated genes and suicidal behavior but reported some interesting findings, such as *rs1130864* in the *CRP* gene associated with suicidal attempts as well as impulsiveness, a well-known risk factor of suicide attempts [113,114]. Other studies reported, although inconclusive, findings regarding the potential association between suicidal phenotypes and variations in *TGFB1* (encoding TGF-β1) [115] *TNFA* (encoding TNF-α) [116,117], *TNF1B* (encoding TNF-1β), *IFNG* (encoding IFN-γ) [117], *IL1*, *IL1B* (encoding IL-1β) and *IL1RN* (encoding Interleukin 1 Receptor Antagonist IL-1RA) [116,118], *IL8* [119], and *IL10* genes [68,117]. More recently, a systematic review of 32 studies investigating associations between suicidal behavior and SNPs in various inflammation-related genes reported that most frequently investigated genes included *TNFα*, *IFNγ*, *ACP1*, *IL1β*, *IL8*, *IL10*, *IL1A*, *IL7*, *TGFB1* and *MIF* (encoding macrophage migration inhibitory factor) and found that polymorphisms in *IL8* (*rs4073*), *CRP* (*rs1130864*), *TNFa* (*rs1800629*, *rs361525*, *rs1099724*), *TNFRII* (*rs1061622*), *TGFB1* (*rs1982073*), *ACP1* (encoding Acid Phosphatase 1) (*rs7419262, rs300774*), *IL10* (*rs1800896*), *IFNG* (*rs2430561*), *ACMSD* (encoding aminocarboxymuconate semialdehyde decarboxylase) (*rs2121337*), *IL7* (*rs10448044*, *rs10448042*), *MIF* (*rs755622*), *IL1A* (*rs1800587*), and *IL1B* (*rs1143634, rs16944*) are likely to be associated with suicidality [120]. However, it must be borne in mind that the reliability and scientific value of candidate gene studies have recently been questioned in part due to their low replicability leading to the abandonment of this approach [121]. GWAS studies also stressed some relevant findings, such as *rs300774* in *ACP1* with a genome-wide significance, and several suggestively significant variants such as *rs10903034* (*IL28RA*), *rs1467558* (*CD44*), *rs6480463* (*ADAMTS14*), *rs4575* (*PSME2*), *rs10448042* and *rs10448044* (*IL7*) [120]. Furthermore, based on data from large GWAS-s, a Mendelian randomization study reported that the upregulation in IL-6 signaling is a potential causal risk factor of suicidality, also suggesting that overactivity of IL-6 may be associated with suicidal behavior, suggesting that IL-6 blockade may be a potential novel treatment or preventive target [64].

Understanding the genetic variations predisposing to such reactions or identifying biomarkers for at-risk profiles may help to detect those individuals who are at a higher risk of progressing towards suicidal behavior under unfavorable environmental or social circumstances.

## 12. Implications of The Role of Cytokines in Suicidal Behavior for Prevention and Treatment

Hopefully, our novel understanding of the involvement of neuroinflammation in suicidal behavior will offer us novel targets and intervention methods. Already several long-used or novel pharmacological molecules are understood to influence immunological processes.

Esketamine inhibits autophagy via mTOR-BDNF signaling pathways, thus alleviating lipopolysaccharide-induced neuroinflammatory and depressive symptoms via reducing levels of cytokines, apoptotic factors, and autophagic markers [122]. Conventional antidepressants, such as SSRIs, also have anti-inflammatory properties, and, in turn, higher levels of inflammation may decrease the efficacy of antidepressant treatment [123]. Lithium is postulated to decrease suicide risk partially by inhibiting glycogen synthase kinase (GSK-3), which is associated with both increased proinflammatory cytokine levels and promoting aggression and depression [124]. Several anti-inflammatory strategies have been reported as being potential treatment options, such as boosting regulatory T-cell response via immunization strategies using CNS antigens attracting T-cells to the brains or through the administration of bacterial and parasitic antigens or the stimulation of the vagus nerve to induce anti-inflammatory acetylcholine-producing T-cells [25,83]. In addition, several currently used nonpsychiatric medications with an effect on neuroinflammation and immune processes may be repurposed to test whether they are effective in reducing suicide risk. For instance, in an epidemiological non-interventional observational study, non-steroidal anti-inflammatory drugs (NSAIDs) were associated with decreased suicide rates [125]. The rest of the potentially repurposable agents have primarily been investigated in depressed patients, as suicidal patients are usually excluded from clinical studies. NSAIDs, as well as cytokine inhibitors, may be able to enhance response to antidepressant medications [126]. Cyclooxigenase-2 inhibitors have been found to be potentially effective as add-on treatments for depression even in a recent meta-analysis of 29 RCTs [127,128,129,130], while their effectiveness in reducing suicide risk has yet to be investigated. TNF-α antibodies, such as infliximab or etanercept, have been reported to decrease both abnormally elevated inflammatory markers and depressive symptoms [131,132,133,134,135,136,137]. IL-6 and IL-6 receptor antibodies are under investigation as well to test their effectiveness for depressive symptoms, with promising preliminary findings [138,139,140]. Minocycline, a broad-range antibiotic, has been shown to decrease microglial activation, and there are ongoing studies using it as an add-on treatment option in various affective disorders [141,142,143,144,145,146]. Finally, pentoxifylline, a phosphodiesterase inhibitor used for vasodilation, has also been found to decrease proinflammatory cytokine expression and depression in animal models [23,147,148,149]. All these agents are currently being tested for repurposing in the treatment of major depression but not suicidal behavior, and it is well known that those patients at increased suicide risk are mostly excluded from clinical studies; thus, it is yet to be determined whether these agents would be effective even in the case of suicide. However, in the case of esketamine, due to its rapid onset of action, there have been studies showing a significant effect on decreasing suicidal ideation. It is yet to be demonstrated whether this is paralleled by its action on proinflammatory processes or on other target points in view of its activity.

There is also evidence that neuroinflammation may characterize only a subset of depressed and suicidal patients, which is supported by the evidence that anti-inflammatory mediators attenuate psychological symptoms only in those showing elevated neuroinflammation [25]. For instance, in one study, the TNF agonist infliximab improved depressive symptoms only in those therapy-resistant depressive patients who showed elevated CRP levels [150]. Such results may also be used towards using anti-inflammatory markers to predict response to treatment. Notably, in another study, baseline CRP levels predicted therapeutic response to both nortriptyline and escitalopram [151]. Such findings also carry the promise of personalizing treatment using inflammatory pathways targeting molecules based on the inflammatory status [68].

Testing whether these molecules and treatments which decrease neuroinflammation are actually effective in depression and suicide will require more time. However, we also have other ways to decrease neuroinflammation in psychiatric patients. Not only medications but psychological therapies have been shown to decrease proinflammatory cytokine levels in individual studies using different psychotherapeutic modalities, such as cognitive–behavioral therapy, supportive depressive dynamic psychotherapy, and mindfulness-based psychotherapeutic interventions. These results were confirmed in a recent meta-analysis of eight different types of psychosocial interventions in 56 RCTs and 4060 participants [152]. These findings also offer us the potential to use cytokine levels as potential markers to monitor the effectiveness of psychotherapies at the molecular level or even to predict who may benefit from such interventions.

In addition to psychotherapies, several other environmental and lifestyle factors may also influence neuroinflammation, such as vitamin D deficiency, the ratio of ingested omega-3/omega-6 fatty acids, loss of sleep, exercise, or restoring gut microbiome. All of these factors are also associated with suicide risk; thus, it is worth investigating whether their effect on cytokines plays a role in modulating their antisuicidal effects.

## 13. Putting Cytokines to Use in Understanding and Managing Suicide Risk: Obstacles and Challenges

At this point in time, we face several difficulties and obstacles in putting our knowledge related to cytokines at the service of suicide prediction and prevention. First, even though repurposed anti-inflammatory molecules may alleviate depressive symptoms, a major obstacle in understanding their effectiveness in reducing suicide risk is that suicidal patients are usually excluded from the majority of clinical trials. Similarly, it requires careful design in clinical studies to separate the association between cytokine and suicidal behavior from their association with depression, especially at the genomic level.

Furthermore, while we have several lines of evidence regarding increased proinflammatory activity and cytokine levels in biological samples of suicide attempters and completers, it is possible that not suicide itself is associated in general with inflammatory processes, but it is characteristic of only a subgroup of patients and a subtype of suicidal behaviors. Given the fortunate scarcity of completed suicide on the population level, it is very difficult, especially in a prospective setting, to gather a sample size with enough power to understand such associations and identify such subgroups.

We also need to gain a much deeper understanding of the involvement of neuroinflammation and proinflammatory cytokines in suicidal behavior and even the specificity of these processes. Considering that cytokines are profoundly involved in fundamental physiological inflammatory processes in the periphery and even in the central nervous system, they have an important role in key neuronal processes, including neurodevelopment, neurodegeneration and beyond, and their manipulation may disturb and cause dysfunctions in critical processes, especially if they are implemented in patients without increased neuroinflammation or neuroinflammation unrelated to suicide risk, thus harming patients via impairing key processes.

Anti-inflammatory drugs also have several off-target effects, which contribute to a large variety of potential adverse effects. For these reasons, drugs that specifically target specific cytokines and pathways in the central nervous systems and their effect on suicide-related targets would be preferable; thus, we need to identify and target further specific signaling and regulatory mechanisms in neuroinflammation [25].

In conclusion, neuroinflammation offers a novel model for emotional, cognitive, and behavioral symptoms and risk in suicidal patients and, thus, novel biomarkers and treatment options. However, for clinical utilization, there is still a long way to proceed with divergent potential next steps. First, we need to better characterize processes of neuroinflammation in suicidal patients, and we need to more deeply characterize suicidal behaviors and specific endophenotypes from a neuroinflammation perspective to identify the directly involved pathways. We should identify specific subgroups of patients who show higher neuroinflammation in parallel to suicide risk, and also those who are genetically more vulnerable to respond with higher neuroinflammation when exposed to certain distal or proximal, inner or outer environmental factors or stressors, but even the specific subtypes or endophenotypes of suicidal behaviors related to neuroinflammation. We should, probably using machine learning and artificial intelligence (AI) methods, develop biomarkers, biomarker profiles as well as signatures to characterize the different subtypes and subgroups. We should also better understand the association between neuroinflammatory markers and the efficacy of psychotherapies to exploit their use for predicting who would be useful candidates for psychotherapy, as well as predict and monitor the effectiveness of psychotherapies. Only after all these steps have been taken will we hopefully find suitable target molecules and novel drug mechanisms supporting the fight against suicide based on neuroinflammation through reliable biomarkers for prediction of risk, selecting effective treatment, and finding novel effective treatments.
